# Social Determinants of Tooth Loss among a Group of Iranian Female Heads of Household

**DOI:** 10.15171/joddd.2015.025

**Published:** 2015-06-10

**Authors:** Taraneh Movahhed, Behjatalmolook Ajami, Mojtaba Dorri, Nima Biouki, Hadi Ghasemi, Mohammad Taghi Shakeri, Mahboobe Dehghani

**Affiliations:** ^1^Assistant Professor of Pediatric Dentistry, Dental Material Research Center, Department of Community Oral Health, School of Dentistry, Mashhad University of Medical Sciences, Mashhad, Iran; ^2^Associate Professor of Pediatric Dentistry, Department of Community Oral Health, School of Dentistry, Mashhad University of Medical Sciences, Mashhad, Iran; ^3^DDS MSc PhD, College London Dental Institute, University College London, London, UK; ^4^Postgraduate student of Oral and Maxillofacial Radiology, School of Dentistry, Tabriz University of Medical Sciences, Tabriz, Iran; ^5^Assistant Professor of Community Dentistry, Department of Community Oral Health, School of Dentistry, Shahid Beheshti University of Medical Sciences, Tehran, Iran; ^6^Associate Professor, School of Medicine, Department of Biostatics, Mashhad University of Medical Sciences, Mashhad, Iran; ^7^Assistant Professor of Orthodontics, Dental Research Center, School of Dentistry, Mashhad University of Medical Sciences, Mashhad, Iran

**Keywords:** Dentition, female, head of household, single-parent family, tooth loss

## Abstract

***Background and aims.*** Tooth loss may lead to mastication disability, which in turn has important impact on individual’s quality of life. Social and psychological factors have been shown to be associated with tooth loss. This study aimed to investigate the social determinants and prevalence of tooth loss, and presence of functional dentition among female heads of household under support of Welfare Organization in Mashhad, Iran.

***Materials and methods.*** In current study 556 participants aged 16-76 years were recruited. Sociodemographic characteristics (age, level of education, family size, and monthly income) were collected using interviewer-led questionnaire. Data about number of teeth and functional dentition were obtained by oral examination. The data were analyzed using Chi-square, Kruskal-Wallis, T-test and binary logistic regression analysis.

***Results.*** Four percent of participants were edentulous. Tooth loss was significantly associated with level of education, age and family size (P < 0.001). There was no significant association between level of income and tooth loss (P = 0.88). Only 37.5% of dentate subjects had functional dentition (anterior and premolar teeth). Women older than 40 years were 0.63 times less likely to have functional dentition than those younger than 40 years. Females with at least a high-school diploma were six times more likely to have functional dentition than their illiterate counterparts.

***Conclusion.*** Social determinants of functional dentition should be taken into account when planning oral health promoting programs for female heads of household. For reducing oral health inequalities access to dental services should be facilitated.

## Introduction

Tooth loss can negatively affect oral health related quality of life.^1^ Studies have indicated different risk factors for tooth loss. Women, those with lower income, with lower educational level, from lower social class and who smoke are more likely to lose their teeth.^2,3,4^

There is association between tooth loss and poor general health. Tu et al^5^ reported a significant association between tooth loss and mortality due to cardiovascular diseases. This may partly be due to impaired nutritional intake as a result of tooth loss and consequently a non-functional dentition.^6^ Studies also showed an increased risk of cognitive decline among older men with higher number of missing teeth.^7^ Michaud et al^8^ in their longitudinal study on the relationship between tooth loss and cancer, with about 18 years follow up period, suggested that those with fewer teeth at baseline (≤16 teeth) versus those with more teeth (25≤teeth≤32) had increased risk of lung cancer.

Although there is association between tooth loss and general health, a full set (32 teeth) of teeth in both arches is not a requirement for a functional dentition. Kayser ^9^ proposed the shortened dental arch concept, that indicates at least four occlusal units preferably in a symmetrical position is adequate for oral function. Kanno & Carlsson^10^ showed that shortened dental arches, comprising anterior and premolar teeth, fulfill the general and basic needs of a functional dentition. Furthermore, World Health Organization indicated maintaining a functional, esthetic, natural dentition of at least 20 teeth, with no need to artificial replacements (prostheses) as the treatment goal for oral health.^11^

The data on the prevalence of tooth loss and functional dentition in the middle aged group is scarce. Studies showed that although only 3% of 35 to 44 year-old Iranians were edentulous, only 76% of dentate subjects had a functional dentition.^12^ Similarly, Mamai-Homata et al^13^ stated that 0.3% of 35-44 year-old Greek adults were edentulous and 91.2% of dentate subjects had 21 teeth or more. Hescot et al^14^ reported that prevalence of edentulism in the same age group in France was 0%. The prevalence edentulous Sudanese adults was reported as 0.1% while 93% of the adults had 21 teeth or more.^15^

In Iran, Welfare Organization is responsible for: support and empowerment of physically and mentally disabled, elderly, female heads of household, orphans and street children, teaching life skills and drug addiction prevention with community-based approach. About 10% of Iranian families had a woman as its household head. The female heads of households usually struggle by low income and lack of social support.^16^ In 2011, the Office for Women and Family at Iranian Welfare Organization announced the number of families supported by this organization to be 3871 households in Mashhad, the capital of Khorasan Province in Eastnorthen Iran. It is important to investigate the factors influencing the functional dentition in this vulnerable disadvantaged community. This will inform strategies to improve oral health in this group of population.

The aim of this study was to assess the social determinants and prevalence of tooth loss and presence of functional dentition among female heads of household supported by the Welfare Organization in Mashhad. We investigated the association between variables including age, educational level, family size, and monthly income with tooth loss and functional dentition status.

## Materials and Methods

Study population included the female heads of household in Mashhad, a city in Northeast of Iran, who were supported by Welfare Organization. The protocol was reviewed and approved by the Research Council of the Mashhad Dental School.

This cross-sectional study was carried out between October and November of 2009. The women participated in the study were heads of their family and responsible for providing the family expenses. Based on the findings of a national study in Iran^12^ which indicated that 76% of 35-44 aged Iranians had functional dentition, the estimated sample size was 437 with 4% chance of error. There are six divisions of Welfare Organization in Mashhad. In this study, volunteer sampling method was used. All the female heads of household in all six divisions were identified by their relevant Welfare Organization division and received an invitation letter. The letter included full description of the study and attendance schedule for oral examination. Subjects with systemic diseases (e.g. diabetes, cancer and cardiovascular diseases) were excluded. 556 women participated in the study and answered an interviewer-led questionnaire with questions on age, level of education, family size, and monthly income. Data about number of teeth and presence or absence of functional dentition were obtained through oral examination. Interviews and oral examinations were conducted by a qualified dentist. Oral examination was carried out on a chair under artificial illumination using sterile dental mirror and explorer. The data collection for each participant took about 10 minutes. All teeth in the mouth were included in the examination except third molar teeth and retained roots. Presence or absence of functional dentition was determined as described by Kanno and Carlsson.^10^ They defined functional dentition as presence of all anterior and premolar teeth in the mouth. Participants who were diagnosed with dental treatment needs were referred to the Mashhad Dental School to receive treatment. Participants also received free toothbrushes and toothpastes to encourage tooth brushing behavior as incentive.

Data analysis was carried out using SPSS software. Data were analyzed using T-test, Kruskal-Wallis, Mann-Whitney, Chi-square, correlation tests (Spearman and Pearson) and logistic regression. Logistic regression model was used to assess the relationship between functional dentition and sociodemographic variables and results were reported as Odds Ratio with 95% confidence interval (CI). Suitability of the regression model was tested with Hosmer-Lemeshow test. P-values equal or less than 0.05 were considered statistically significant.

## Results

The mean age was 40.45 ± 9.6. Four percent of female heads of household were edentulous. Older women; those with lower educational level; and with more than four family members were more likely to be edentulous. Frequency distribution of dentate subjects based on sociodemographic factors are presented in Table 1.

**Table 1:** Frequency distribution of dentate subjects based on sociodemographic factors.

**Factor**	**Women Percent**	**Number of Teeth**				**P value**	**r**
		**1-9 10-19 20-24 25-28**					
**Dentate**	96	3.9	28.5	28.8	38.8		
**Age groups**							
16-34	31	0	10.3	20.6	69.1	<0.0001	_0.56
35-44	35.6	2.6	26.5	38.1	26.6		
45-54	27.7	8.8	42.9	26.6	19.7		
≥55years	5.7	10	63.3	20	6.7		
**Income (Rials)**							
≤870000	50.7	3.4	26.8	26.4	43.4	0.88	+0.008
88-2080000	39.6	4.3	30.4	32.9	32.4		
≥2090000	9.7	3.9	29.4	29.4	37.3		
**Size of family**							
≤4	63.3	4.7	34.3	29.4	31.6	<0.0001	_0.16
>4	36.7	3.3	25.4	28.8	42.5		
**Educational level**							
Illiterate	14.9	7.9	58.4	25.8	7.9	<0.0001	+0.41
Less than Diploma	64.6	4.2	27.1	29.5	39.2		
Diploma or higher	20.5	1.1	7.4	28.4	64.2		

Only 37.5 % of dentate subjects and 35.5% of all subjects had functional dentition. Older subjects were significantly less likely to have functional dentition than younger subjects (P < 0.001; Table 2). There was positive significant association between level of education and functional dentition (P < 0.001), so that presence of functional dentition was more probable in more educated women ( Table 2). Functional dentition was also associated with size of family (P = 0.004) and income (P = 0.035; Table 2).

**Table 2:** Assessment the effect of each sociodemographic factor separately on presence of functional dentition.

**Factor**	**Number of Teeth**	**P value**
	**Present not present**	
**Age groups**				
16-34	62	38	p<0.001
35-44	35.1	64.9	
45-54	18.7	81.3	
≥55years	13.3	86.7	
**Income (Rials)**				
≤870000	42.1	57.9	p=0.035
88-2080000	32.8	67.2	
>2090000	31.4	68.6	
**Size of family**			
≤4	39.8	60.2	p=0.004
>4	32.3	67.7	
**Educational level**				
Illiterate	12.8	87.2	p<0.001
Less than Diploma	35.1	64.9	
Diploma or higher	61.2	38.8	

Logistic regression model was used for assessment of the simultaneous effect of sociodemographic factors on the presence of functional dentition. In simultaneous effect assessment, only age and level of education were significantly associated with the presence of functional dentition. Women older than 40 years were 0.63 times less likely to have functional dentition than those younger than 40 years ( Table 3). Females with at least Diploma were six times more likely to have functional dentition than their illiterate counterparts ( Table 3).

**Table 3:** The mean, minimum and maximum difference between the BL dimension of the teeth and that of SSCs (mm)

**Factor**	**Estimates**	**SE**	**OR**	**P value**	**95% CI**
**Age(0=age≤40,1=age>40)**	_0.98	0.198	0.37	<0.001	0.25-0.55
**Income**	0.00	0.00	1	0.45	1.00-1.00
**Size of family**	_0.035	0.05	0.96	0.54	0.86-1.08
**Educational level (0=illiterate,4=diploma and higher)**	+1.84	0.37	6.32	<0.0001	3.03-13.19
Goodness-of-fit: Hosmer–Lemeshow test = 0. 23					
OR: odds ratios; CI: confidence intervals					

Among studied factors just age of dentate women showed significant relation with prosthodontic rehabilitation (upper jaw: P <0.0001; lower jaw: P = 0.016). Prosthodontic rehabilitation was more prevalent in older age groups. Prevalence of prosthodontic rehabilitation in upper jaw was more than lower jaw.

## Discussion

In this cross-sectional study tooth loss and presence of functional dentition frequency among Mashhad female heads of household who were under support of Welfare Organization was investigated.

Small proportion of the study sample (4%) was edentulous. This was even less prevalent among 35-44 years old women (2 %). In the age group above 55 years old, 23% of women were edentulous, but number of women in this age group was low. Whilst 3% of 35-44 year old Iranians were reported as edentulous, this was less prevalent among Greeks (0.3%) and French (0%).^12-14^ However, the participants in these studies were different. Subjects in the present study were female heads of household while the Greek and French studies were carried out among the general population. There is lack of data on the prevalence of tooth loss among female heads of household in other communities, which in turn did not allow us to compare this parameter among different countries. However high prevalence of tooth loss among our study population compared to other communities suggests poorer oral health among Iranian female heads of household.

More than a third (38.1%) of 35-44 year-old dentate subjects in the current study had 20-24 teeth and about a quarter (26.6%) had 25-28 teeth (Table 1). In total, only 64.7% of 35-44 year-old dentate subjects had 20 or more teeth, whilst it is reported that 76% of 35-44 year-old dentate Iranians have 20 or more teeth.^12^ This suggests that the frequency of having 20 or more teeth is lower in female heads of household compared to general population in Iran. This indicates that this group of the society needs further attention when planning oral health promoting programs. WHO recommended that 90% of 35-44 year-olds should have at least 20 teeth by 2010.^17^

There was a strong association between higher level of education and presence of functional dentition. This is in line with findings from other studies that showed association between higher levels of education and protection against tooth loss ^12,15,18^ De Marchi et al evaluated the psychosocial factors involved on tooth loss among an old age population.^4^ Participants illustrated that lack of public dental health policies and programs, and low level of oral health knowledge have a major role on tooth loss prevalence.

In the present study presence of functional dentition among women over 40 years was 63% less likely than younger women. Similarly, other studies indicated the association between age and tooth loss.^19,12^

In this study presence of more teeth was more likely among families with 4 members or less (Table 1). Mucci et al^21^ showed that increasing the number of family members increases the odds of tooth loss by 10%. It may be partly explained that smaller families will have more resources for education and also providing healthy food and oral hygiene devices. However the studies in this area are so limited and further research is recommended.

There was no significant relationship between the number of teeth and monthly family income. This disagrees with findings from other studies. Newton et al^22^ indicated that income had direct effect on oral health behavior, psychosocial stress and oral health. Pallegedara et al^20^ and Hamasha et al^23^ reported that level of income was significantly correlated with the number of remaining teeth. This disagreement may be due to that 90.6 % of women in the present study had monthly income lower than poverty line (209 × 104 Rials). ^24^ Although government programs have targeted to reduce the number of families below the poverty line, poverty continues to be a major social problem. However in our study income didn’t show association with presence of functional dentition or teeth retention. It may be due to that the level of income in all three groups was low and with these income levels, people could not spend enough for dental care. In other words almost all of studied subjects were near or under the poverty line and we could not assess the effect of monthly income on presence of functional dentition truly.

High prevalence of tooth loss among the female heads of household may be partly due to poor access of this group to dental services.

## Conclusion

According to our study only about one-third of female heads of household residing in Mashhad had functional dentition and tooth loss prevalence was high. Factors influencing functional dentition should be taken into account when planning oral health promoting programs for female heads of household. Furthermore, access to dental services should be facilitated through enhancing collaboration between Welfare Organization, oral health Office of Ministry of Health and Medical Education, and community dentistry department of dental schools.
